# Supplementary material for: Mental health outcomes and intimate partner violence among nepalese women: A propensity score matched study
Source: PLOS Ment Health. 2025 Jul 10;2(7):e0000374. doi: 10.1371/journal.pmen.0000374 (PMC12798303; doi:10.1371/journal.pmen.0000374)
Supplement: S1 Table — (DOCX) [file pmen.0000374.s001.docx]

**S1 Table** Nepal anxiety and depression cut-off scores for adolescent and adult population.

|  | **Cut-off score** | |
| --- | --- | --- |
| **Tool** | Adolescents(up to 19 years | Adults (20 years and above) |
| GAD-7 | 7 or more | 9 or more |
| PHQ-9 | 11 or more | 10 or more |
