# Supplementary material for: Mental health outcomes and intimate partner violence among nepalese women: A propensity score matched study
Source: PLOS Ment Health. 2025 Jul 10;2(7):e0000374. doi: 10.1371/journal.pmen.0000374 (PMC12798303; doi:10.1371/journal.pmen.0000374)
Supplement: S2 Table — (DOCX) [file pmen.0000374.s002.docx]

**S2 Table:** Potential confounders used in the study

| **Confounder variable** | **Variable type as recorded in 2022 NDHS** | **Description** | **Variable type used in the study** |
| --- | --- | --- | --- |
| Age | Continuous and Categorical | Age in years of the respondent | Categorical (15-24, 25-34, 35-49) |
| Education level | Categorical | Highest level of education of the survey respondent | Categorical (None, Basic, Secondary/Higher) |
| Region | Categorical | The first level administrative regions (provinces) of Nepal where the respondent resided at the time of the survey. | Categorical (Bagmati, Gandaki, Karnali, Koshi, Lumbini, Madhesh, Sudurpashchim) |
| Food insecurity | Categorical | Moderate or severe food insecurity experience by respondent or a member of the household using food insecurity experience scale (FIES) developed by Food and Agriculture Organization (FAO). FIES elicits self-reported experiences and behaviors related to food access due to lack of money or other resources, over a 12-month recall period, irrespective of frequency of occurrence. FIES is comprised of 8 questions ranging in the severity of food insecurity they measure, from low food insecurity (question 1) to severe food insecurity (question 8). Respondents answer yes/no to the 8 questions and the responses are aggregated to give raw scores ranging from 0 to 8. Food insecurity was classified into 3 categories: 1) food secure, with raw scores = 0–3; 2) moderate, with raw scores = 4–6; and 3) Severe, with raw scores = 7–8. | Categorical (Yes for moderate or severe food insecurity/No) |
| Self-reported health status | Categorical | Self-reported health status by respondent | Categorical (bad, moderate, good) |
| Substance / | Categorical | Use of tobacco or alcohol ( more than 4 bottles per week) | Categorical (Yes/No) |
| Alcohol use by partner | Categorical | Alcohol use by respondent’s current or former intimate partner | Categorical (Yes/No) |
| Pregnancy/child loss | Categorical | If respondent ever lost a child or pregnancy | Categorical (Yes/No) |
| Severe disability | Categorical | Severe disability was defined as having a lot of difficulties in performing daily activities or unable to do anything at all. | Categorical (Yes/No) |
| Income status | Categorical | Respondent’s income status whether seasonal, none or all year round | Categorical (None, seasonal, all year round) |
| Marital status | Categorical | Respondent’s marital status | Categorical (Single, married/cohabiting, divorced/widowed) |
