# Supplementary material for: Mental health outcomes and intimate partner violence among nepalese women: A propensity score matched study
Source: PLOS Ment Health. 2025 Jul 10;2(7):e0000374. doi: 10.1371/journal.pmen.0000374 (PMC12798303; doi:10.1371/journal.pmen.0000374)
Supplement: S3 Table — (DOCX) [file pmen.0000374.s003.docx]

**S3 Table** Social demographic characteristics of the study population.

|  | | **Total(N=4245)** | |
| --- | --- | --- | --- |
| **Variable** | **Category** | **Number(n)** | **Proportion (%)** |
| Age | 15-24 | 1,011 | 23.8 |
|  | 25-34 | 1,521 | 35.8 |
|  | 35-49 | 1,712 | 40.3 |
| Education | Basic | 1,371 | 32.3 |
|  | No education | 1,315 | 31.0 |
|  | Secondary or Higher | 1,559 | 36.7 |
| Region | Bagmati | 837 | 19.7 |
|  | Gandaki | 417 | 9.8 |
|  | Karnali | 272 | 6.4 |
|  | Koshi | 728 | 17.1 |
|  | Lumbini | 753 | 17.7 |
|  | Madhesh | 871 | 20.5 |
|  | Sudurpashchim | 367 | 8.6 |
| Marital | Married/living with partner | 3,853 | 90.8 |
|  | Single | 214 | 5.0 |
|  | Widowed/Separated | 178 | 4.2 |
| Health | Bad | 436 | 10.3 |
|  | Good | 1,391 | 32.8 |
|  | Moderate | 2,418 | 57.0 |
| Income | All year | 2,025 | 47.7 |
|  | None | 999 | 23.5 |
|  | Seasonal | 1,221 | 28.8 |
| Substance | No | 3,855 | 90.8 |
|  | Yes | 390 | 9.2 |
| Pregnancy/child loss | No | 2,922 | 68.8 |
|  | Yes | 1,323 | 31.2 |
| Severe disability | No | 3,953 | 94.2 |
|  | Yes | 242 | 5.8 |
| Food insecurity | No | 2,596 | 61.2 |
|  | Yes | 1,645 | 38.8 |
| Partner drinks | No | 2,069 | 48.8 |
|  | Yes | 2,175 | 51.2 |
