# Supplementary material for: Mental health outcomes and intimate partner violence among nepalese women: A propensity score matched study
Source: PLOS Ment Health. 2025 Jul 10;2(7):e0000374. doi: 10.1371/journal.pmen.0000374 (PMC12798303; doi:10.1371/journal.pmen.0000374)
Supplement: S4 Table — (DOCX) [file pmen.0000374.s004.docx]

**S4 Table** Survey weighted prevalence estimates of the individual types of IPVs and their co-occurrence with male controlling behavior in the unmatched sample.

| **Type of IPV** | **Prevalence—no. (%)** | **(95% CI)** |
| --- | --- | --- |
| Any IPV | 1264 ( 29.8 ) | 28.4 - 31.2 |
| Physical IPV only | 156 ( 12.3 ) | 10.6 - 14.3 |
| Emotional IPV only | 98 ( 7.8 ) | 6.4 - 9.4 |
| Sexual IPV only | * | * |
| Controlling behavior only | 548 ( 43.4 ) | 40.6 - 46.1 |
| Physical IPV and controlling behavior | 216 ( 17.1 ) | 15.1 - 19.3 |
| Emotional IPV and controlling behavior | 239 ( 18.9 ) | 16.8 - 21.2 |
| Sexual IPV and controlling behavior | 107 ( 8.5 ) | 7.1 - 10.1 |

___________________

****Indicates estimates based on <25 unweighted samples; these values have been suppressed***
