# Supplementary material for: Mental health outcomes and intimate partner violence among nepalese women: A propensity score matched study
Source: PLOS Ment Health. 2025 Jul 10;2(7):e0000374. doi: 10.1371/journal.pmen.0000374 (PMC12798303; doi:10.1371/journal.pmen.0000374)
Supplement: S5 Table — (DOCX) [file pmen.0000374.s005.docx]

**S5 Table** Survey weighted prevalence estimates of symptoms of generalized anxiety and depression by type of IPV.

| **Exposure** | **Outcome** | **Prevalence—no. (%)** | **95% CI** |
| --- | --- | --- | --- |
| Any IPV | Symptoms of anxiety or depression | 283 ( 22.4 ) | 20.2 - 24.8 |
|  | Symptoms of anxiety | 145 ( 11.5 ) | 9.8 - 13.4 |
|  | Symptoms of depression | 138 ( 10.9 ) | 9.3 - 12.8 |
| Physical IPV | Symptoms of anxiety or depression | * | * |
|  | Symptoms of anxiety | * | * |
|  | Symptoms of depression | * | * |
| Emotional IPV | Symptoms of anxiety or depression | 25 ( 25.5 ) | 17.9 – 35.0 |
|  | Symptoms of anxiety | * | * |
|  | Symptoms of depression | * | * |
| Sexual IPV | Symptoms of anxiety or depression | * | * |
|  | Symptoms of anxiety | * | * |
|  | Symptoms of depression | * | * |
| Controlling behavior | Symptoms of anxiety or depression | 86 ( 15.7 ) | 12.9 – 19.0 |
|  | Symptoms of anxiety | 53 ( 9.7 ) | 7.5 - 12.4 |
|  | Symptoms of depression | 33 ( 6.0 ) | 4.3 - 8.4 |
| Physical IPV and controlling behavior | Symptoms of anxiety or depression | 78 ( 35.9 ) | 29.9 - 42.5 |
|  | Symptoms of anxiety | 26 ( 12.0 ) | 8.3 – 17.0 |
|  | Symptoms of depression | 52 ( 24.1 ) | 18.9 - 30.2 |
| Emotional IPV and controlling behavior | Symptoms of anxiety or depression | 104 ( 43.5 ) | 37.4 - 49.9 |
|  | Symptoms of anxiety | 40 ( 16.7 ) | 12.5 – 22.0 |
|  | Symptoms of depression | 64 ( 26.8 ) | 21.6 - 32.7 |
| Sexual IPV and controlling behavior | Symptoms of anxiety or depression | 44 ( 41.1 ) | 32.3 - 50.6 |
|  | Symptoms of anxiety | * | * |
|  | Symptoms of depression | 33 ( 30.8 ) | 22.9 - 40.1 |

___________________

****Indicates estimates based on <25 unweighted samples; these values have been suppressed.***
