# Supplementary material for: Mental health outcomes and intimate partner violence among nepalese women: A propensity score matched study
Source: PLOS Ment Health. 2025 Jul 10;2(7):e0000374. doi: 10.1371/journal.pmen.0000374 (PMC12798303; doi:10.1371/journal.pmen.0000374)
Supplement: S6 Table — (DOCX) [file pmen.0000374.s006.docx]

**S6 Table** Covariate balance for physical violence co-occurring with male controlling behavior

|  | **Unmatched** | | | **PS Matched** | | |
| --- | --- | --- | --- | --- | --- | --- |
| **Characteristic** | **Unexposed**  **(**3201) | **Exposed**  **(220**) | **ASMD** | **Unexposed (220**) | **Exposed (220**) | **ASMD** |
| **Age—no. (%)** |  |  |  |  |  |  |
| 15-24 | 599 (18.7) | 47 (21.4) | 0.211 | 42 (19.1) | 47 (21.4) | 0.075 |
| 25-34 | 1230 (38.4) | 101 (45.9) |  | 99 (45.0) | 101 (45.9) |  |
| 35-49 | 1372 (42.9) | 72 (32.7) |  | 79 (35.9) | 72 (32.7) |  |
| **Education—no. (%)** |  |  |  |  |  |  |
| Basic | 1084 (33.9) | 72 (32.7) | 0.295 | 72 (32.7) | 72 (32.7) | 0.057 |
| No education | 999 (31.2) | 96 (43.6) |  | 91 (41.4) | 96 (43.6) |  |
| Secondary or Higher | 1118 (34.9) | 52 (23.6) |  | 57 (25.9) | 52 (23.6) |  |
| **Health status—no. (%)** |  |  |  |  |  |  |
| Bad | 353 (11.0) | 37 (16.8) | 0.207 | 37 (16.8) | 37 (16.8) | <0.001 |
| Good | 913 (28.5) | 70 (31.8) |  | 70 (31.8) | 70 (31.8) |  |
| Moderate | 1935 (60.4) | 113 (51.4) |  | 113 (51.4) | 113 (51.4) |  |
| **Income status—no. (%)** |  |  |  |  |  |  |
| All year | 1702 (53.2) | 96 (43.6) | 0.192 | 98 (44.5) | 96 (43.6) | 0.021 |
| None | 559 (17.5) | 45 (20.5) |  | 45 (20.5) | 45 (20.5) |  |
| Seasonal | 940 (29.4) | 79 (35.9) |  | 77 (35.0) | 79 (35.9) |  |
| **Region—no. (%)** |  |  |  |  |  |  |
| Bagmati | 500 (15.6) | 19 ( 8.6) | 0.495 | 19 ( 8.6) | 19 ( 8.6) | 0.071 |
| Gandaki | 424 (13.2) | 19 ( 8.6) |  | 18 ( 8.2) | 19 ( 8.6) |  |
| Karnali | 461 (14.4) | 21 ( 9.5) |  | 24 (10.9) | 21 ( 9.5) |  |
| Koshi | 475 (14.8) | 31 (14.1) |  | 33 (15.0) | 31 (14.1) |  |
| Lumbini | 453 (14.2) | 46 (20.9) |  | 43 (19.5) | 46 (20.9) |  |
| Madhesh | 423 (13.2) | 62 (28.2) |  | 59 (26.8) | 62 (28.2) |  |
| Sudurpashchim | 465 (14.5) | 22 (10.0) |  | 24 (10.9) | 22 (10.0) |  |
| **Marital status—no. (%)** |  |  |  |  |  |  |
| Married/living with partner | 2961 (92.5) | 216 (98.2) | 0.294 | 216 (98.2) | 216 (98.2) | <0.001 |
| Single | 88 ( 2.7) | 0 ( 0.0) |  | 0 ( 0.0) | 0 ( 0.0) |  |
| Widowed/Separated | 152 ( 4.7) | 4 ( 1.8) |  | 4 ( 1.8) | 4 ( 1.8) |  |
| **Partner drinks—no. (%)** |  |  |  |  |  |  |
| No | 1637 (51.1) | 50 (22.7) | 0.616 | 50 (22.7) | 50 (22.7) | <0.001 |
| Yes | 1564 (48.9) | 170 (77.3) |  | 170 (77.3) | 170 (77.3) |  |
| **Substance use—no. (%)** |  |  |  |  |  |  |
| No | 2862 (89.4) | 188 (85.5) | 0.120 | 188 (85.5) | 188 (85.5) | <0.001 |
| Yes | 339 (10.6) | 32 (14.5) |  | 32 (14.5) | 32 (14.5) |  |
| **Pregnancy/child loss—no. (%)** |  |  |  |  |  |  |
| No | 2151 (67.2) | 127 (57.7) | 0.197 | 131 (59.5) | 127 (57.7) | 0.037 |
| Yes | 1050 (32.8) | 93 (42.3) |  | 89 (40.5) | 93 (42.3) |  |
| **Severe disability—no. (%)** |  |  |  |  |  |  |
| No | 3005 (93.9) | 207 (94.1) | 0.009 | 208 (94.5) | 207 (94.1) | 0.020 |
| Yes | 196 ( 6.1) | 13 ( 5.9) |  | 12 ( 5.5) | 13 ( 5.9) |  |
| **Food insecurity—no. (%)** |  |  |  |  |  |  |
| No | 2649 (82.8) | 147 (66.8) | 0.373 | 147 (66.8) | 147 (66.8) | <0.001 |
| Yes | 552 (17.2) | 73 (33.2) |  | 73 (33.2) | 73 (33.2) |  |
