# Supplementary material for: Mental health outcomes and intimate partner violence among nepalese women: A propensity score matched study
Source: PLOS Ment Health. 2025 Jul 10;2(7):e0000374. doi: 10.1371/journal.pmen.0000374 (PMC12798303; doi:10.1371/journal.pmen.0000374)
Supplement: S8 Table — (DOCX) [file pmen.0000374.s008.docx]

**S8 Table** Covariate balance table for sexual violence co-occurring with male controlling behavior

|  | **Unmatched** | | | **PS Matched** | | |
| --- | --- | --- | --- | --- | --- | --- |
| **Characteristic** | **Unexposed**  **(**3201) | **Exposed**  **(**111) | **SMD** | **Unexposed**  **(**111) | **Exposed**  **(**111) | **SMD** |
| **Age—no. (%)** |  |  |  |  |  |  |
| 15-24 | 599 (18.7) | 26 (23.4) | 0.168 | 24 (21.6) | 26 (23.4) | 0.058 |
| 25-34 | 1230 (38.4) | 46 (41.4) |  | 49 (44.1) | 46 (41.4) |  |
| 35-49 | 1372 (42.9) | 39 (35.1) |  | 38 (34.2) | 39 (35.1) |  |
| **Education—no. (%)** |  |  |  |  |  |  |
| Basic | 1084 (33.9) | 48 (43.2) | 0.249 | 48 (43.2) | 48 (43.2) | 0.024 |
| No education | 999 (31.2) | 36 (32.4) |  | 37 (33.3) | 36 (32.4) |  |
| Secondary or Higher | 1118 (34.9) | 27 (24.3) |  | 26 (23.4) | 27 (24.3) |  |
| **Health status—no. (%)** |  |  |  |  |  |  |
| Bad | 353 (11.0) | 23 (20.7) | 0.268 | 23 (20.7) | 23 (20.7) | <0.001 |
| Good | 913 (28.5) | 28 (25.2) |  | 28 (25.2) | 28 (25.2) |  |
| Moderate | 1935 (60.4) | 60 (54.1) |  | 60 (54.1) | 60 (54.1) |  |
| **Income status—no. (%)** |  |  |  |  |  |  |
| all year | 1702 (53.2) | 55 (49.5) | 0.174 | 54 (48.6) | 55 (49.5) | 0.027 |
| no income | 559 (17.5) | 15 (13.5) |  | 16 (14.4) | 15 (13.5) |  |
| Seasonal | 940 (29.4) | 41 (36.9) |  | 41 (36.9) | 41 (36.9) |  |
| **Region—no. (%)** |  |  |  |  |  |  |
| Bagmati | 500 (15.6) | 12 (10.8) | 0.385 | 13 (11.7) | 12 (10.8) | 0.058 |
| Gandaki | 424 (13.2) | 12 (10.8) |  | 11 ( 9.9) | 12 (10.8) |  |
| Karnali | 461 (14.4) | 14 (12.6) |  | 15 (13.5) | 14 (12.6) |  |
| Koshi | 475 (14.8) | 20 (18.0) |  | 21 (18.9) | 20 (18.0) |  |
| Lumbini | 453 (14.2) | 19 (17.1) |  | 18 (16.2) | 19 (17.1) |  |
| Madhesh | 423 (13.2) | 26 (23.4) |  | 25 (22.5) | 26 (23.4) |  |
| Sudurpashchim | 465 (14.5) | 8 ( 7.2) |  | 8 ( 7.2) | 8 ( 7.2) |  |
| **Marital status—no. (%)** |  |  |  |  |  |  |
| Married/living with partner | 2961 (92.5) | 108 (97.3) | 0.264 | 108 (97.3) | 108 (97.3) | <0.001 |
| Single | 88 ( 2.7) | 0 ( 0.0) |  | 0 ( 0.0) | 0 ( 0.0) |  |
| Widowed/Separated | 152 ( 4.7) | 3 ( 2.7) |  | 3 ( 2.7) | 3 ( 2.7) |  |
| **Partner drinks—no. (%)** |  |  |  |  |  |  |
| No | 1637 (51.1) | 27 (24.3) | 0.576 | 27 (24.3) | 27 (24.3) | <0.001 |
| Yes | 1564 (48.9) | 84 (75.7) |  | 84 (75.7) | 84 (75.7) |  |
| **Substance use—no. (%)** |  |  |  |  |  |  |
| No | 2862 (89.4) | 96 (86.5) | 0.090 | 96 (86.5) | 96 (86.5) | <0.001 |
| Yes | 339 (10.6) | 15 (13.5) |  | 15 (13.5) | 15 (13.5) |  |
| **Pregnancy/child loss—no. (%)** |  |  |  |  |  |  |
| No | 2151 (67.2) | 60 (54.1) | 0.271 | 62 (55.9) | 60 (54.1) | 0.036 |
| Yes | 1050 (32.8) | 51 (45.9) |  | 49 (44.1) | 51 (45.9) |  |
| **Severe disability—no. (%)** |  |  |  |  |  |  |
| No | 3005 (93.9) | 103 (92.8) | 0.043 | 103 (92.8) | 103 (92.8) | <0.001 |
| Yes | 196 ( 6.1) | 8 ( 7.2) |  | 8 ( 7.2) | 8 ( 7.2) |  |
| **Food insecurity—no. (%)** |  |  |  |  |  |  |
| No | 2649 (82.8) | 75 (67.6) | 0.357 | 75 (67.6) | 75 (67.6) | <0.001 |
| Yes | 552 (17.2) | 36 (32.4) |  | 36 (32.4) | 36 (32.4) |  |
