# Supplementary material for: Mental health outcomes and intimate partner violence among nepalese women: A propensity score matched study
Source: PLOS Ment Health. 2025 Jul 10;2(7):e0000374. doi: 10.1371/journal.pmen.0000374 (PMC12798303; doi:10.1371/journal.pmen.0000374)
Supplement: S9 Table — (DOCX) [file pmen.0000374.s009.docx]

**S9 Table** Covariate balance for any violence excluding women who gave birth 12 months prior to the survey

|  | **Unmatched** | | | **PS Matched** | | |
| --- | --- | --- | --- | --- | --- | --- |
| **Characteristic** | **Unexposed**  **(**3198) | **Exposed**  **(**95) | **ASMD** | **Unexposed (**95) | **Exposed (**95) | **ASMD** |
| **Age—no. (%)** |  |  |  |  |  |  |
| 15-24 | 599 (18.7) | 25 (26.3) | 0.237 | 26 (27.4) | 25 (26.3) | 0.026 |
| 25-34 | 1227 (38.4) | 39 (41.1) |  | 38 (40.0) | 39 (41.1) |  |
| 35-49 | 1372 (42.9) | 31 (32.6) |  | 31 (32.6) | 31 (32.6) |  |
| **Education—no. (%)** |  |  |  |  |  |  |
| Basic | 1083 (33.9) | 36 (37.9) | 0.140 | 35 (36.8) | 36 (37.9) | 0.044 |
| No education | 999 (31.2) | 32 (33.7) |  | 34 (35.8) | 32 (33.7) |  |
| Secondary or Higher | 1116 (34.9) | 27 (28.4) |  | 26 (27.4) | 27 (28.4) |  |
| **Health status—no. (%)** |  |  |  |  |  |  |
| Bad | 353 (11.0) | 17 (17.9) | 0.279 | 17 (17.9) | 17 (17.9) | <0.001 |
| Good | 912 (28.5) | 33 (34.7) |  | 33 (34.7) | 33 (34.7) |  |
| Moderate | 1933 (60.4) | 45 (47.4) |  | 45 (47.4) | 45 (47.4) |  |
| **Income status—no. (%)** |  |  |  |  |  |  |
| all year | 1700 (53.2) | 36 (37.9) | 0.310 | 38 (40.0) | 36 (37.9) | 0.088 |
| no income | 559 (17.5) | 22 (23.2) |  | 24 (25.3) | 22 (23.2) |  |
| Seasonal | 939 (29.4) | 37 (38.9) |  | 33 (34.7) | 37 (38.9) |  |
| **Region—no. (%)** |  |  |  |  |  |  |
| Bagmati | 498 (15.6) | 6 ( 6.3) | 0.645 | 6 ( 6.3) | 6 ( 6.3) | 0.086 |
| Gandaki | 424 (13.3) | 8 ( 8.4) |  | 10 (10.5) | 8 ( 8.4) |  |
| Karnali | 460 (14.4) | 8 ( 8.4) |  | 8 ( 8.4) | 8 ( 8.4) |  |
| Koshi | 475 (14.9) | 14 (14.7) |  | 13 (13.7) | 14 (14.7) |  |
| Lumbini | 453 (14.2) | 15 (15.8) |  | 16 (16.8) | 15 (15.8) |  |
| Madhesh | 423 (13.2) | 35 (36.8) |  | 33 (34.7) | 35 (36.8) |  |
| Sudurpashchim | 465 (14.5) | 9 ( 9.5) |  | 9 ( 9.5) | 9 ( 9.5) |  |
| **Marital status—no. (%)** |  |  |  |  |  |  |
| Married/living with partner | 2958 (92.5) | 89 (93.7) | 0.253 | 89 (93.7) | 89 (93.7) | <0.001 |
| Single | 88 ( 2.8) | 5 ( 5.3) |  | 5 ( 5.3) | 5 ( 5.3) |  |
| Widowed/Separated | 152 ( 4.8) | 1 ( 1.1) |  | 1 ( 1.1) | 1 ( 1.1) |  |
| **Partner drinks—no. (%)** |  |  |  |  |  |  |
| No | 1637 (51.2) | 33 (34.7) | 0.337 | 34 (35.8) | 33 (34.7) | 0.022 |
| Yes | 1561 (48.8) | 62 (65.3) |  | 61 (64.2) | 62 (65.3) |  |
| **Substance use—no. (%)** |  |  |  |  |  |  |
| No | 2859 (89.4) | 82 (86.3) | 0.095 | 84 (88.4) | 82 (86.3) | 0.063 |
| Yes | 339 (10.6) | 13 (13.7) |  | 11 (11.6) | 13 (13.7) |  |
| **Pregnancy/child loss—no. (%)** |  |  |  |  |  |  |
| No | 2149 (67.2) | 60 (63.2) | 0.085 | 61 (64.2) | 60 (63.2) | 0.022 |
| Yes | 1049 (32.8) | 35 (36.8) |  | 34 (35.8) | 35 (36.8) |  |
| **Severe disability—no. (%)** |  |  |  |  |  |  |
| No | 3002 (93.9) | 86 (90.5) | 0.125 | 87 (91.6) | 86 (90.5) | 0.037 |
| Yes | 196 ( 6.1) | 9 ( 9.5) |  | 8 ( 8.4) | 9 ( 9.5) |  |
| **Food insecurity—no. (%)** |  |  |  |  |  |  |
| No | 1915 (59.9) | 47 (49.5) | 0.210 | 47 (49.5) | 47 (49.5) | <0.001 |
| Yes | 1283 (40.1) | 48 (50.5) |  | 48 (50.5) | 48 (50.5) |  |
