# Supplementary material for: Mental health outcomes and intimate partner violence among nepalese women: A propensity score matched study
Source: PLOS Ment Health. 2025 Jul 10;2(7):e0000374. doi: 10.1371/journal.pmen.0000374 (PMC12798303; doi:10.1371/journal.pmen.0000374)
Supplement: S10 Table — (DOCX) [file pmen.0000374.s010.docx]

**S10 Table** PSM univariable and multivariale modified Poisson regression outcome analysis results for women exposed to any IPV using Nepal GAD-7 and PHQ-9 Cut-off values.

| **Exposure** | **Outcome** | **Univariable model** | | | **Multivariable model** | | |
| --- | --- | --- | --- | --- | --- | --- | --- |
|  |  | **RR** | **95% CI** | **P Value** | **aRR** | **95%CI** | **P Value** |
| Any violence | Symptoms of anxiety  or depression | 2.18 | 1.81-2.63 | <0.001 | 2.01 | 1.66-2.42 | <0.001 |
|  | Symptoms of anxiety | 1.70 | 1.3-2.23 | <0.001 | 1.55 | 1.17-2.05 | 0.002 |
|  | Symptoms of depression | 2.67 | 2-3.55 | <0.001 | 2.56 | 1.92-3.40 | <0.001 |
