# Supplementary material for: Mental health outcomes and intimate partner violence among nepalese women: A propensity score matched study
Source: PLOS Ment Health. 2025 Jul 10;2(7):e0000374. doi: 10.1371/journal.pmen.0000374 (PMC12798303; doi:10.1371/journal.pmen.0000374)
Supplement: S12 Table — (DOCX) [file pmen.0000374.s012.docx]

**S12 Table** Univariable and multivariable modified Poisson regression analysis results for women exposed to any IPV for the unmatched sample.

| **Exposure** | **Outcome** | **Univariable model** | | | **Multivariable model** | | |
| --- | --- | --- | --- | --- | --- | --- | --- |
|  |  | **RR** | **95% CI** | **P Value** | **aRR** | **95%CI** | **P Value** |
| Any violence | Symptoms of anxiety  or depression | 2.05 | 1.73-2.43 | <0.001 | 1.88 | 1.57-2.25 | <0.001 |
|  | Symptoms of anxiety | 1.68 | 1.32-2.13 | <0.001 | 1.51 | 1.17-1.96 | 0.002 |
|  | Symptoms of depression | 2.68 | 2.01-3.56 | <0.001 | 2.56 | 1.92-3.40 | <0.001 |
