# Supplementary material for: Mental health outcomes and intimate partner violence among nepalese women: A propensity score matched study
Source: PLOS Ment Health. 2025 Jul 10;2(7):e0000374. doi: 10.1371/journal.pmen.0000374 (PMC12798303; doi:10.1371/journal.pmen.0000374)
Supplement: S13 Table — (DOCX) [file pmen.0000374.s013.docx]

**S13 Table** E-values for point estimates and lower 95% confidence limits for outcomes for which there was observed associations with IPV.

|  |  | **aRR** | **95%CI** | E-Value for point estimate | E-Value* lower 95%CI |
| --- | --- | --- | --- | --- | --- |
| Any violence | Symptoms of anxiety  or depression | 1.88 | 1.57-2.25 | 3.12 | 2.52 |
|  | Symptoms of anxiety | 1.51 | 1.17-1.96 | 2.39 | 1.62 |
|  | Symptoms of depression | 2.56 | 1.92-3.40 | 4.56 | 3.25 |
| Emotional IPV | Symptoms of depression | 2.42 | 1.09-5.36 | 4.27 | 1.40 |
| Physical IPV and controlling bahaviours | Symptoms of anxiety  or depression | 3.07 | 2.04-4.62 | 5.59 | 3.50 |
|  | Symptoms of depression | 4.74 | 2.54-8.83 | 8.95 | 4.52 |
| Emotional IPV and controlling bahaviours | Symptoms of anxiety  or depression | 3.07 | 2.10-4.49 | 5.59 | 3.62 |
|  | Symptoms of anxiety | 1.93 | 1.06-3.51 | 3.27 | 1.31 |
|  | Symptoms of depression | 4.98 | 2.75-9.01 | 9.43 | 4.94 |
| Sexual IPV and Controlling behaviors | Symptoms of anxiety  or depression | 3.13 | 2.31-4.24 | 5.71 | 4.05 |
|  | Symptoms of depression | 7.00 | 4.52-10.85 | 13.85 | 8.51 |
