# Supplementary material for: Mental health outcomes and intimate partner violence among nepalese women: A propensity score matched study
Source: PLOS Ment Health. 2025 Jul 10;2(7):e0000374. doi: 10.1371/journal.pmen.0000374 (PMC12798303; doi:10.1371/journal.pmen.0000374)
Supplement: Attachment — Submitted filename: Response to Reviewers.docx [file pmen.0000374.s015.docx]

**Mental Health Outcomes and Intimate Partner Violence among Nepalese Women: A Propensity Score Matched Study**

Reviewer Comments:

**Reviewer 1:**

-     The authors are affiliated with institutions in Kenya, was there anyone from Nepal involved as this is usually good practice to understand the context.

**Response:** We thank the reviewer for raising this important question about the study context. While Nepalese co-investigation would have been ideal, the research team has extensive experience with these data. Our study team has previously conducted a study using the same nationally representative survey dataset, providing in-depth knowledge of the Nepalese context[1]. Furthermore, although initial attempts to collaborate with researchers in Nepal for this study were unsuccessful, we conducted a comprehensive literature review to ensure the validity of our findings' interpretation.

- Were any weights used to adjust for non-response etc? Please outline what they accounted for.
What was done to deal with missing data? What was the response rate for the survey?

**Response:** We thank the reviewer for the comments and provide an itemized response below:

-Were any weights used to adjust for non-response etc? Please outline what they accounted for.

**Response:** We thank the reviewer for the comment. We clarify that the DHS sampling weights explicitly include adjustment for non-response in addition to unequal selection probabilities [2].

- What was done to deal with missing data?

**Response:** We thank the reviewer for the comment. We have included the following statement in **Lines 199-200:**

“Given the low proportion of missing data (less than 1.4%), we did not perform multiple imputation. “

- What was the response rate for the survey?

**Response:** We thank the reviewer for the comment. We have included the following statement in **Line 111-114:**

“Female interviewers invited all eligible women aged 15 to 49 from selected households to participate in face-to-face interviews, ensuring a nationally representative sample of households and women of reproductive age. Of the 15, 238 eligible women 14, 845 completed the interviews, resulting in a 97.4% response rate [2].”

- Typo in Figure 1 red box – information
-Line 273 reference error

**Response:** We thank the reviewer for flagging the error. These errors have been corrected in Figure 1.

-Line 241 - refers to 1 person exposed to sexual violence. I don't know if the DHS has any rules about reporting small numbers, but the authors may want to reconsider reporting this due to potential sensitivities

**Response:** We thank the reviewer for the important observation. We have excluded this proportion from our report. Furthermore, for clarity, we included a statement.

**Lines: 244-245**: “Estimates based on fewer than 25 unweighted samples were excluded due to concerns about statistical reliability, following DHS reporting guidelines.”[3]

**Reviewer 2:**

The study uses a cross-sectional design, which limits its ability to determine cause and effect. The propensity score matching only controls for variables that were measured, leaving room for unaccounted confounders. Self-reported data on IPV and mental health can lead to underreporting and recall bias. The small sample size for certain IPV types reduces the power to detect significant associations. Overall, these methodological issues weaken the reliability of the study’s conclusions. Addressing these issues can significantly improve the study.

**Response:** We thank the reviewer for these comments. We respond to each comment as follows:

-The study uses a cross-sectional design, which limits its ability to determine cause and effect. The propensity score matching only controls for variables that were measured, leaving room for unaccounted confounders.

**Response:** As the reviewer notes, cross-sectional studies have inherent limitations. To address this, we have removed a potentially misleading statement about causality from the discussion section and provided a more detailed limitations section to guide the reader's interpretation of our findings. The statement now reads as follows, **Lines 346-348**:

“Our analyses relied on cross-sectional survey data, which inherently limits our ability to determine the temporal sequence between IPV and mental health outcomes, making it challenging to definitively establish the causal direction.”

-The propensity score matching only controls for variables that were measured, leaving room for unaccounted confounders.

We agree with the reviewer that our study design cannot fully exclude potential confounding and highlighted it as an important limitation. However, we performed comprehensive sensitivity analyses to assess the robustness of our findings. These included assessing the effect of unmeasured confounding using E-values. Our results demonstrated that any unmeasured confounder ought to be strongly (>=2.39) associated with both the outcomes and exposures to fully nullify the observed associations. Furthermore, a comprehensive literature review did not establish any variables with such a strong and plausible association.

**Result section** **Lines 274-282**: “To assess the robustness of our findings against unmeasured confounding, E-values for each exposure and corresponding mental health outcomes for which statistically significant associations were observed were calculated (Supp. Table 13). Among women experiencing any form of IPV, the E-value was 3.12, indicating that unmeasured confounding would need to be strongly associated with both IPV and generalized anxiety or depression to account for the observed magnitude of risk. Similarly, the E-values of any IPV were substantial (≥2.39 for anxiety, ≥4.56 for depression) when anxiety and depression were analyzed separately. These results were consistent in the subgroup analyses involving individual types of IPV and their co-occurrence with intimate partner controlling behavior, E-values > 3 (Supp. Table 13).”

**Discussion section** **Lines 337-343**: “In addition, despite the robust methodology deployed (PSM), the possibility of unmeasured confounding cannot be entirely ruled out. However, the E-value analysis suggests that any such confounder would need a markedly strong association with both IPV exposures and mental health outcomes, with a relative risk exceeding 2, to significantly impact the observed findings. Furthermore, a comprehensive literature review did not establish any variables with such a strong and plausible association.”

- The small sample size for certain IPV types reduces the power to detect significant associations.

**Response:** We agree with the reviewer that the sample size is an important limitation. We have highlighted that in the limitations section. **Lines 361-365**:

“Finally, sub-setting the data to create clean cases and control groups can inherently reduce statistical power, potentially leading to an increased risk of type II errors. Therefore, the lack of statistically significant associations between physical IPV and male controlling behaviors, excluding co-occurrence, with mental health outcomes may be attributable, in part, to reduced statistical power.”

References

1.	Barini G, Amima S, Mulwa D, Mogeni P. Spatial heterogeneity of menstrual discriminatory practices against Nepalese women: A population-based study using the 2022 Demographic and Health Survey. PLOS Global Public Health. 2024 Nov 13;4(11):e0003145.

2.	ICF NM of H and P New ERA and. Nepal Demographic and Health Survey 2022. 2023 Jun 15 [cited 2023 Nov 11]; Available from: https://dhsprogram.com/publications/publication-FR379-DHS-Final-Reports.cfm

3.	D’Anna L. Analyzing DHS Data [Internet]. [cited 2025 Apr 20]. Available from: https://dhsprogram.com/data/Guide-to-DHS-Statistics/Analyzing_DHS_Data.htm
