# Supplementary material for: Mental health outcomes and intimate partner violence among nepalese women: A propensity score matched study
Source: PLOS Ment Health. 2025 Jul 10;2(7):e0000374. doi: 10.1371/journal.pmen.0000374 (PMC12798303; doi:10.1371/journal.pmen.0000374)
Supplement: Attachment — Submitted filename: Response_to_Reviewers_auresp_2.docx [file pmen.0000374.s016.docx]

**Mental Health Outcomes and Intimate Partner Violence among Nepalese Women: A Propensity Score Matched Study**

Reviewer Comments:

**Reviewer 1:**

-    I have a minor suggestion for the abstract - to include full numerical results for the figures given in the final sentence of the findings section as already provided for the other numerical results.

**Response:** We thank the reviewer for the suggestion. We have included the following statement in the abstract **Lines 48-51:**

“However, the associations with generalized anxiety symptoms were modest across all forms of IPV co-occurring with male controlling behavior. That is, aRR=1.84; ([95%CI: 0.87-3.90]; p=0.111) for Physical IPV, aRR=1.93; ([95%CI: 1.06-3.51]; p=0.032) for Emotional IPV, and aRR=1.21; ([95%CI: 0.61-2.39, p=0.588]) for Sexual IPV.”

-   On Line 264 the link has an error.

**Response**: We thank the reviewer for the observation. We have corrected the error. The cross-ref link is now in **line 277.**

**Reviewer 3:**

**Response:** We thank the reviewer for the suggestion. We have rephrased the section to read as follows **lines 379-382**

“**Funding source**

No funding was received for these analyses, writing, or publication. The corresponding author had full access to the dataset and had final responsibility for the decision to submit the manuscript for publication. The DHS had no role in data analysis, interpretation of results, or manuscript writing.”

**Reviewer 4:**

**Response:** We thank the reviewer for the comment. We have included the results of E-value analysis**.**

In the methods section, we have included the following statement **Lines 38-40**

“The modified Poisson regression was used to assess the risk of anxiety or depression in women exposed to IPV, and the E-values were used to assess sensitivity to unmeasured confounding.”

In the result section of the abstract, we have added the following statement in **Lines 51-53**

“The E-value analysis suggests that an unmeasured confounder would need a strong association (aRR>2.39) with both IPV and mental health outcomes to negate the observed findings. “
